# A second species of *Oculogryphus* (Coleoptera, Lampyridae), with notes on the phylogenetic affinities of the genus

**DOI:** 10.3897/zookeys.97.1223

**Published:** 2011-05-11

**Authors:** Ming-Luen Jeng, Marc A. Branham, Michael S. Engel

**Affiliations:** 1 Division of Entomology, Natural History Museum and Department of Ecology and Evolutionary Biology, 1501 Crestline Drive-Suite 140, University of Kansas, Lawrence, Kansas 66049–2811, U.S.A.; 2 Zoology Department, National Museum of Natural Sciences, No. 1, Guanqian Rd., Taichung City 40453, Taiwan, R.O.C.; 3 Department of Entomology and Nematology, PO Box 110620, Bldg. 970, Natural Area Drive, University of Florida, Gainesville, Florida 32611, U.S.A.

**Keywords:** *Oculogryphus*, Lampyridae, phylogenetic position, *Stenocladius*, Vietnam, taxonomy

## Abstract

A second species of the enigmatic lampyrid genus *Oculogryphus* is described and figured as *Oculogryphus bicolor* **sp. n**. from Vietnam. The definition of the genus is slightly modified with consideration of newly detected morphological variation from this species. According to a comprehensive phylogenetic analysis including nearly 80% of documented lampyrid genera, *Oculogryphus* is the putative sister group to *Stenocladius* s. str. within the paraphyletic group of Ototretinae-Ototretadrilinae.The classification of *Stenocladius* is briefly discussed in this context.

## Introduction

The genus *Oculogryphus* was established for a single male specimen of a peculiar species, *Oculogryphus fulvus* Jeng, from Vietnam (Jeng et al. 2007). A second species of the genus, also from Vietnam, was recently identified while examining material in the American Museum of Natural History (AMNH). Herein we describe and figure this new species and discuss some morphological variations between it and *Oculogryphus fulvus*, requiring a slight modification to the generic diagnosis. In addition, comments are provided regarding the phylogenetic position of *Oculogryphus* in the light of a comprehensive phylogenetic analysis of Lampyridae conducted by the senior author (Jeng 2008).

## Material and methods

The methodology and morphological terminology used herein follows that of Jeng et al. (2007). The body length (BL) is the sum of the pronotal and elytral lengths (PL and EL, respectively) plus length of those exposed portions of the head from the pronotum; while body width is considered as twice the elytral width (BW = 2EW). Pronotal width was abbreviated as PW. The nomenclature of the hind wing venation follows that of Kukalová-Peck and Lawrence (2004) rather than that of Kukalová-Peck and Lawrence (1993) which was employed by Jeng et al. (2007). In reporting label data the symbol “/” indicates separate lines on a single labe, while “//” denotes material located on separate labels.

## Results

### Genus Oculogryphus

Jeng, Engel & Yang, 2007

http://species-id.net/wiki/Oculogryphus

Oculogryphus  Jeng, Engel & Yang, 2007: 4.

#### Type-species.

*Oculogryphus fulvus* Jeng, 2007, by original designation.

#### Diagnosis.

The original diagnosis of the genus provided by Jeng et al. (2007) was based strictly on *Oculogryphus fulvus*. The genus is characterized by its large and ventrally approximate compound eyes which are clearly emarginate posteriorly, filiform antennae, unmodified mandibles (*sensu* Green 1959), considerably exposed head from pronotum, absence of tibial spurs and lanterns, progressively shortened tarsomeres from I–IV, eight abdominal ventrites which are not lobed, dorsal abdominal spiracles not enclosed by parasternites, symmetrical aedeagal sheath and aedeagi, and several other characters (Jeng et al. 2007). The new species demonstrates some variations in hind wing venation (bifurcate MP3+4). The following pairs contrast the different hind wing venations under the two nomenclatural systems used between Jeng et al. (2007) and the current work: CuA = Cu; CuA1+2 = CuA; AA = AA3+4; CuA3+4 + AA = AA3. Otherwise the original definition of the genus remains unchanged. These modifications do not affect the key to Oriental genera provided by Jeng et al*.* (2007).

### Oculogryphus bicolor sp. n.

urn:lsid:zoobank.org:act:649F93C0-8F6F-46A8-A163-040DB4AFA3CE

http://species-id.net/wiki/Oculogryphus_bicolor

Figs 1 [Fig F2] 6 

#### Holotype.

♂, “VIETNAM: Ha Tinh, Huong/ Son, 18°22'N, 106°13'E/ 900m, April 20–28, 1998/ Malaise, AMNH, Carpenter/ Grimaldi, Herman, Silva, Long”. Deposited in the Division of Invertebrate Zoology (Entomology), American Museum of Natural History, NY, with eventual deposition in the Institute of Ecology and Biological Resources Collection (IEBR), Hanoi, Vietnam.

#### Paratypes.

4 ♂♂, with identical data as holotype; 1 ♂, with identical data except collected on 18 May 1998; 1 ♂, with identical data as holotype except collected at 600m above sea level on 7–14 April 1998 by K. Long; 1 ♂, with identical data as holotype except collected on 5 May 1998 by K. Long. All deposited in the AMNH.

#### Type-locality.

Vietnam, Ha Tinh Province, Huong Son, 18°22'N, 106°13'E.

#### Diagnosis.

The species has several diagnosable characters separating it from the type species: 1) body size slightly larger (6.2–8.2 vs. 6.0 mm); 2) more vivid light brown-tan bicoloration (Fig. 1); 3) slightly broader elytral epipleura (Fig. 2); 4) bifurcate MP3+4 in the hind wing (Fig. 3); 5) more slender metatibia (Fig. 4); and, 6) more elongate parameres in the male genitalia (Fig. 6).

**Figure:**
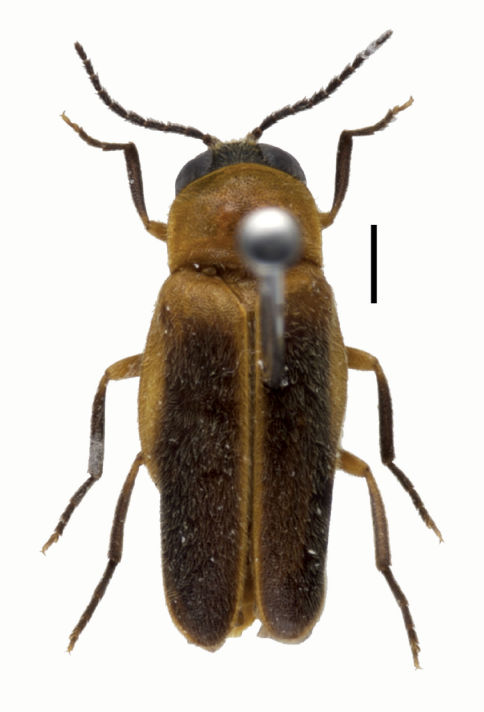
**Figure 1.** Habitus of male of *Oculogryphus bicolor* sp. n. Scale bar = 1.5mm. Elytra somewhat twisted in outer margin of apical half due to dehydration.

**Figure F2:**
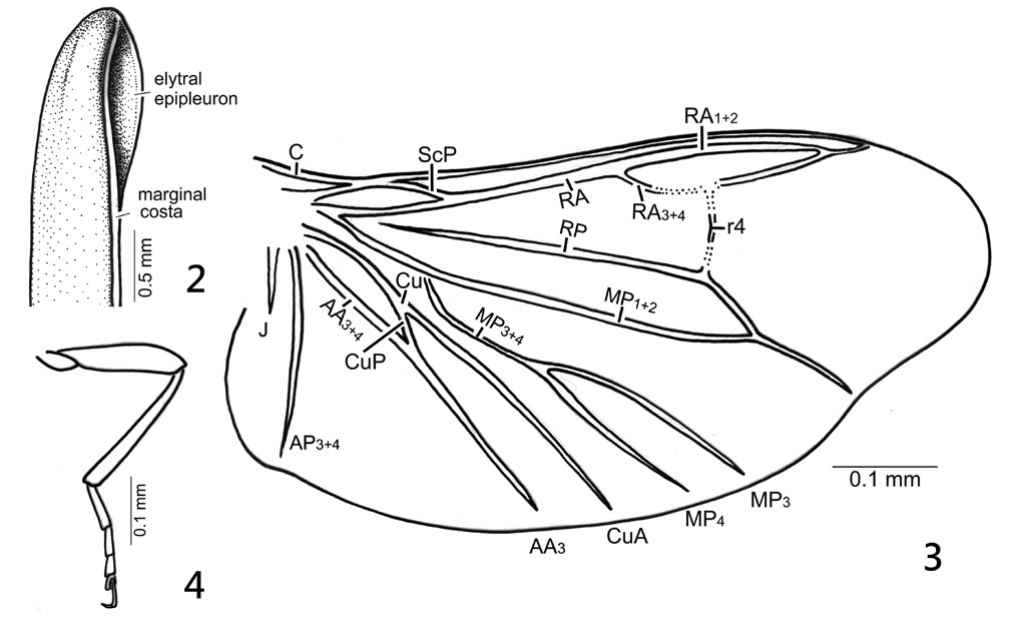
**Figures 2–4.** *Oculogryphus bicolor* sp. n., male **2** basal part of right elytron, lateral aspect, showing epipleuron **3** right hind wing **4** left metatibia, from trochanter to pretarsal claws, ventral aspect.

**Figure:**
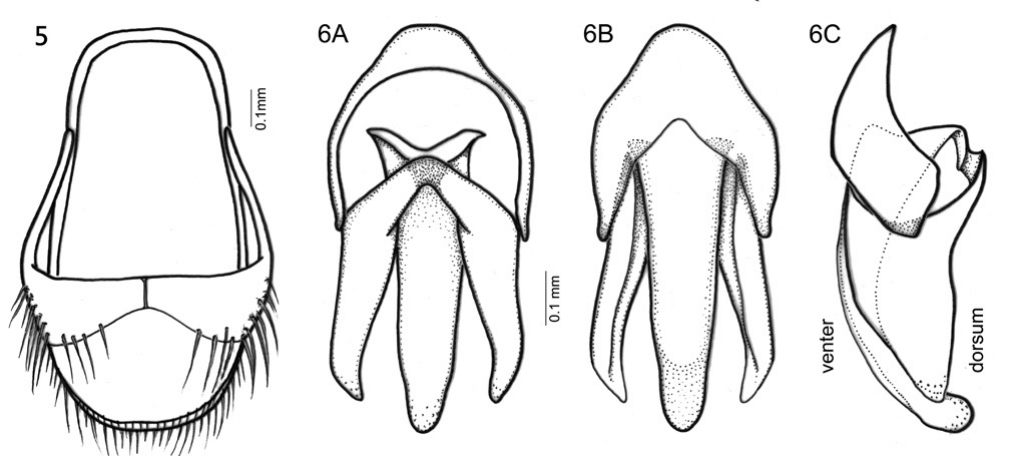
**Figures 5–6.** *Oculogryphus bicolor* sp. n., male **5** aedeagal sheath, dorsal aspect **6** male genitalia, dorsal (A), ventral (B), and lateral (C) aspects.

#### Description.

Male. BL: 6.2–8.2 mm; BW: 2.7–3.4 mm; PW/PL = 1.5–1.6; EL/ EW = 3.6–3.8; EL/PL = 3.8–4.4; BW/PW = 1.4–1.5. The species is very similar to the type species in general morphology except the aforementioned differences, and need not be repeated here. As described for *Oculogryphus fulvus* except: aedeagal sheath has length of 0.9 mm and width of 0.6 mm; abdominal tergites IX and X clearly recognizable individually (Fig. 5); aedeagus about 0.75 mm in length and 0.4 mm broad; parameres comparatively elongate, about as long as basal piece laterally (Fig. 6).

#### Etymology.

The specific epithet refers to the two-toned coloration which is more vivid in the new species than in *Oculogryphus fulvus*.

#### Phenology.

According to the available collection data, males appear at least from April to May.

### Oculogryphus fulvus

Jeng

http://species-id.net/wiki/Oculogryphus_fulvus

Oculogryphus fulvus  Jeng, *in* Jeng, Engel and Yang, 2007: 7.

#### Comments.

A second specimen of *Oculogryphus fulvus*, deposited in Magyar Természettudományi Múzeum, Budapest, was discovered. No significant variation was detected between this specimen and the holotype for the species. Refer to Jeng et al. (2007) for a complete account of this species.

#### New material.

♂, “Vietnam: Cuc Phuong/ Ninh binh, 3-10.V.1966. Exp. Gy. TOPÁL// Nr. 261/ beaten from bushes”.

##### Key to Oculogryphus species

**Table:** 

1	Elytra vividly bicolor, with base, lateral margins, and sutures yellowish brown and disc dark brown; body size larger (body length 6.2–8.2 mm); male genitalia with median lobe slightly surpassing apex of parameres	*Oculogryphus bicolor* sp. n.
–	Elytra more or less uniformly brown in coloration; body size smaller (body length 6.0 mm); male genitalia with median lobe far surpassing apex of parameres by about 1/3 length of median lobe	*Oculogryphus fulvus*

## Discussion

*Oculogryphus* has a mosaic of features intermingling those of Rhagophthalmidae (e.g., large and posteriorly emarginate compound eyes), Luciolinae (e.g., large head partially exposed from pronotum; filiform antennae), Ototretinae (e.g., similar morphology of abdomen; symmetrical aedeagal sheath and aedeagus; absence of lanterns), Ototretadrilinae (e.g., abdominal spiracles not enclosed by parasternites), and Lampyrinae (e.g., ventrally approximate compound eyes) (Jeng et al. 2007). Based on their morphological comparison across 80 documented genera of Lampyridae, Jenget al. (2007) inferred that the genus was either a member of Ototretinae or a stem taxon linking Luciolinae and some basal lineages of Lampyridae. It now appears that this inference was quite accurate. In the most comprehensive phylogenetic analysis of Lampyridae conducted to date (Jeng 2008, in prep.), *Oculogryphus* was found to be the sister group of *Stenocladius* Fairmaire within the paraphyletic group of Ototretinae-Ototretadrilinae complex (both subfamilies were defined by Crowson 1972). Morphologically this position appears intuitive, and seems further supported by the discovery of *Oculogryphus bicolor* which shows an even more similar morphology with that of *Stenocladius*, especially in the aedeagal sheath and male genitalia (*cf*. Kawashima 1999). However, *Stenocladius* is a problematic taxon. The type species, *Stenocladius davidis* Fairmaire from China, has pectinate antennae bearing branches arising from the base of flagellar articles I–VIII, and with flagellar articles that are not abbreviated. All *Stenocladius* species from Japan, Taiwan, and adjacent islands share a more-or-less uniform male genital structure (Kawashima 1999). By contrast, some species of *Stenocladius* are questionably assigned to this genus, especially those from India and Sri Lanka [e.g., *Stenocladius basalis* Pic which was the only representative of *Stenocladius* examined by Crowson (1972) when establishing his new subfamilial classification for Lampyridae]. We have termed the former group as *Stenocladius* s. str. so as to differentiate them from those atypical group(s) within the genus. Several significant differences have been detected among these atypical species, such as the position of the abdominal spiracles (enclosed by parasternites rather than adjacent to them), a different kind of pectinate antennae (abbreviated flagellar articles and branches arising from the article apex rather than the base), and male genitalia (short median lobe and parameres somewhat hooked inward apically or subapically). It is likely that many of the Oriental *Stenocladius* do not truly belong to this genus but are allied to some other ototretines. Further differentiation of *Stenocladius* is under investigation by the senior author. Regardless, the discovery of enigmatic new taxa like *Oculogryphus bicolor* and the recognition of significant difficulties in the current classification of some diverse genera, highlights the need for continued exploration and taxonomic study of Asian Lampyridae.

## Supplementary Material

XML Treatment for Oculogryphus

XML Treatment for Oculogryphus bicolor

XML Treatment for Oculogryphus fulvus
